# Resistance Rates of *Mycobacterium tuberculosis* Complex Strains: A Retrospective Study in Türkiye

**DOI:** 10.3390/medicina61061060

**Published:** 2025-06-09

**Authors:** Melda Payaslıoğlu, İmran Sağlık, Cüneyt Özakın

**Affiliations:** Department of Medical Microbiology, Faculty of Medicine, Bursa Uludağ University, Bursa 16000, Turkey; imransaglik@uludag.edu.tr (İ.S.); ozakin@uludag.edu.tr (C.Ö.)

**Keywords:** *Mycobacterium tuberculosis*, multidrug resistance, Türkiye

## Abstract

*Background and Objectives*: Tuberculosis (TB) is one of the most common infectious diseases in developing countries. The resistance of the causative agent, *Mycobacterium tuberculosis*, to two or more first-line anti-TB drugs results in multidrug-resistant (MDR) TB, posing a serious challenge to the control of TB worldwide. This study was designed to determine the changes in drug resistance over time in TB strains isolated from patients in all departments of Uludağ University Hospital in western Türkiye. *Materials and Methods*: We retrospectively analyzed 104,598 clinical samples sent to our laboratory for the investigation of the presence of TB between 1996 and 2023. BACTEC 460 TB, BACTEC MGIT 960 culture systems and Löwenstein–Jensen medium were used for the culture of these samples. The susceptibility of *M. tuberculosis* complex strains grown in culture to isoniazid (INH) (0.1 μg/mL), rifampicin (RIF) (1.0 μg/mL), ethambutol (ETB) (5.0 μg/mL) and streptomycin (SM) (1.0 μg/mL) antibiotics was studied according to the manufacturer’s recommendation. *Results*: Out of 104,598 patient samples, 2752 (2.6%) were culture-positive, and the susceptibility test results of 1869 of these were analyzed. Of the isolates, 358 (19.2%) were found to be resistant to at least one first-line drug, i.e., INH, RIF, ETB, or SM. In addition, 2.9% were resistant to two or more first-line drugs. *Conclusions*: Drug susceptibility testing is essential to ensure the optimal treatment and control of drug-resistant TB strains. This study highlights the value of ongoing efforts to control tuberculosis drug resistance in the fight against this disease.

## 1. Introduction

Tuberculosis (TB) is a chronic granulomatous infectious disease caused by *Mycobacterium tuberculosis* complex. Although the World Health Organization (WHO) has reported that the incidence of the disease has decreased worldwide in recent years, the goals of the WHO TB elimination program have not yet been achieved due to several factors, including the increase in human immunodeficiency virus (HIV) infections, increased migration, and deficiencies in public health system infrastructure [1]. Isoniazid (INH), rifampicin (RIF), ethambutol (ETM), and streptomycin (SM) are used as first-line TB drugs. Declared a threat to global health by the WHO in 1993, TB continues to be a major infectious disease worldwide; mortality rates due to increasing resistance to the antibiotics used in treatment are projected to equal cancer-related mortality rates by 2050 [1,2,3]. The resistance of *M. tuberculosis* to two or more anti-TB drugs, especially INH and RIF, is referred to as multidrug-resistant (MDR) TB, and these resistant strains pose a serious challenge to controlling TB worldwide [1,2,3,4,5,6,7,8,9,10,11]. Worldwide, MDR-TB and/or RIF-resistant TB were detected in an estimated 3.2% of new cases and 16.0% of previously treated cases in 2023 [3]. In this context, TB programs continue to plan to combat the emergence and consequences of drug resistance to anti-TB drugs. The most important obstacles to eliminating TB are delayed diagnosis and the spread of drug-resistant TB cases. Therefore, the early diagnosis of TB cases and testing drug susceptibility are important for the care of TB patients and the control of this disease [1]. It has been reported that there is a significant difference between the estimated number of MDR-positive TB cases and the number reported to WHO; it is reported that in 2023, an estimated 44% of cases were diagnosed and treated as MDR TB, while 66% of cases were undetected [2,3].

Since TB prevalence and resistant strain rates may vary between regions and over time, epidemiological information on regional anti-TB resistance rates is important in the global TB fight.

This study aimed to retrospectively evaluate the susceptibility of TB strains to first-line anti-TB drugs (INH, RIF, ETM and SM) among TB strains isolated from Uludağ University Hospital in Bursa province, located in western Türkiye, between 1996 and 2023. Although there are many studies on MDR TB strains, our study is very important in terms of examining the changes in resistance over a very long period of 28 years, comparing these data with our country’s data and showing the impact of the implementation of WHO recommendations in the fight against TB.

## 2. Materials and Methods

### 2.1. Samples

In this study, the results of 104,598 samples sent to our laboratory with a preliminary diagnosis of TB between 1 January 1996 and 31 December 2023 were evaluated retrospectively. During this 28-year period, patients who applied to various clinics and polyclinics of our hospital, which serves as a tertiary health institution in Bursa and accepts patients from surrounding provinces, and who were suspected of pulmonary or extrapulmonary TB as a result of clinical evaluation and examinations were included in the study. Mycobacteriology laboratory reports from this period were examined retrospectively. Respiratory system samples (sputum, bronchial lavage and bronchoalveolar lavage) and non-respiratory system samples (paracentesis fluid, urine, cerebrospinal fluid, wound discharge, joint fluid and abscess) sent to our mycobacteriology laboratory for the investigation of the presence of TB were evaluated. Direct microscopic examination (smear) and culture tests were applied to all samples; the susceptibilities of *M. tuberculosis* complex strains grown in culture to first-line TB drugs (INH, RIF, ETM, SM) were examined. Since the susceptibility results of repeated samples from the same patient could affect the resistance rates and the analysis of this subject, they were excluded from the evaluation and 1878 susceptibility results were analyzed. The mycobacteriology laboratory where the study was conducted is included in the External Quality Assessment Program of the Turkish Public Health Agency.

### 2.2. Culture, Identification and Anti-TB Susceptibility Testing of Mycobacteria

A total of 104,598 clinical samples taken from patients with a preliminary diagnosis of TB were cultured according to Löwenstein–Jensen medium procedures; the BACTEC 460 TB (Becton Dickinson Diagnostic, Franklin Lakes, NJ, USA) culture system was used for samples between 1996 and 2003, and the BACTEC 960 MGIT (Becton Dickinson Diagnostic, USA) culture system was used for samples between 2004 and 2023. For mycobacteria typing, p-Nitro-α-acetylamino-β-hydroxypropiophenone (NAP) was used in the BACTEC 460 TB system, and p-nitrobenzoic acid (pNBA) was used in the BACTEC 960 MGIT system and the BD MGITM TBc Identification Test (Becton Dickinson Diagnostic, USA) kit. This study did not aim to compare the differences in positivity between the LJ and MGIT methods. Positivity in either method was considered sufficient for anti-TB tests and the results were analyzed accordingly. In the tests of susceptibility to major anti-TB drugs, INH, RIF, ETB, SM were applied at concentrations of 1.0, 0.1, 5.0 and 1.0 µg/mL, respectively, in accordance with the operating procedures of the systems.

### 2.3. Statistical Analysis

In the statistical analysis, four-year time periods were used instead of annual intervals to prevent short-term fluctuations on an annual basis from overshadowing general trends and to ensure that changes in the dataset over time could be analyzed correctly.

Since our aim was to determine a general increase or decrease trend over time, only one trend was focused on, separate comparisons were not made between matched groups, and multiple test correlations were not required.

Descriptive statistics are presented as frequency and percentage. The Chi-square trend test was used to evaluate changes in the culture positivity and MDR rates over time. This test is a suitable method for analyzing possible linear trends in rates. In our study, the statistical significance of trends over time was evaluated with this test, and the odds ratio (OR) values were given. Statistically, the significance level was accepted as α = 0.05. Statistical analyses were performed with IBM SPSS 29.0.2.0 (IBM Corp. Released 2023. IBM SPSS Statistics for Windows, Version 29.0.2.0 Armonk, NY, USA: IBM Corp.).

## 3. Results

The anatomical distribution and positivity rates of a total of 104,598 samples analyzed for the presence of TB between 1996 and 2023, separated according to whether they were pulmonary (respiratory tract samples) (52.1%) or extrapulmonary (47.9%), are presented in Table 1. When the distribution of a total of 2752 samples in which *M. tuberculosis* positivity was detected during the study was examined, it was determined that 1858 (68.2%) samples were of pulmonary origin and 874 (31.8%) samples were of extrapulmonary origin.

In this study, it was found that the culture positivity rates, which were 2.6% in all samples, varied between 1.5% and 4.8% in the 1996–2023 period and showed a statistically significant decreasing trend over the years (*p* < 0.001). In our study, it can be seen that the culture positivity rate is low (2.6%) due to the evaluation of a large-scale sample sent with clinical suspicion instead of a general screening of the population.

**Figure 1:**
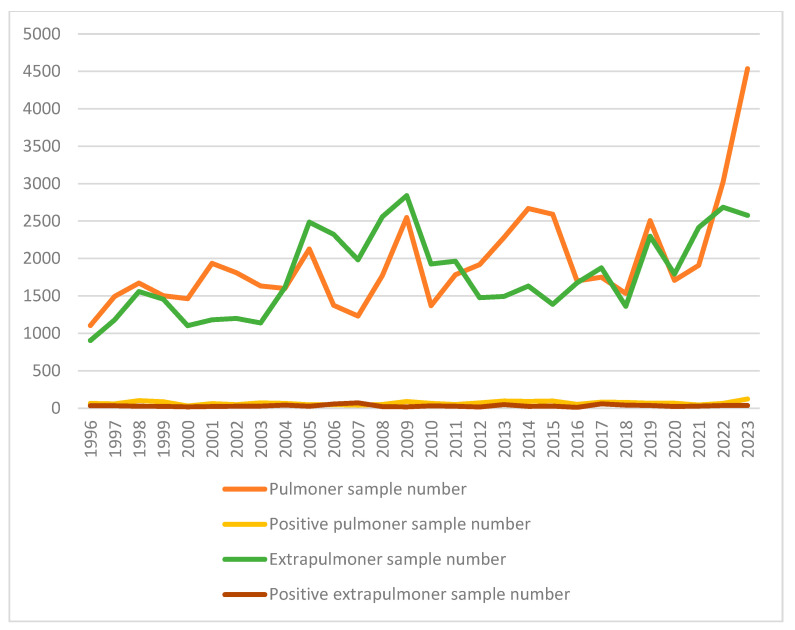
Change in the number of samples examined by year (1996–2023).

When the data were grouped into 4-year periods, a statistically significant decreasing trend was observed in the positivity rates of both pulmonary and extrapulmonary samples during the study period (pulmonary: *p* < 0.001; extrapulmonary: *p* < 0.001). However, a significant increase in the positivity of extrapulmonary samples was observed during the 2004–2007 period (Table 2, Figure 1). The distribution of the anti-TB drug susceptibility of the 1869 *M. tuberculosis* strains included in the study by years is shown in Table 3. In general, 358 (19.2%) of the isolates were found to be resistant to at least one first-line drug, and 2.9% (54 strains) were found to be resistant to two or more first-line drugs.

**Figure 2:**
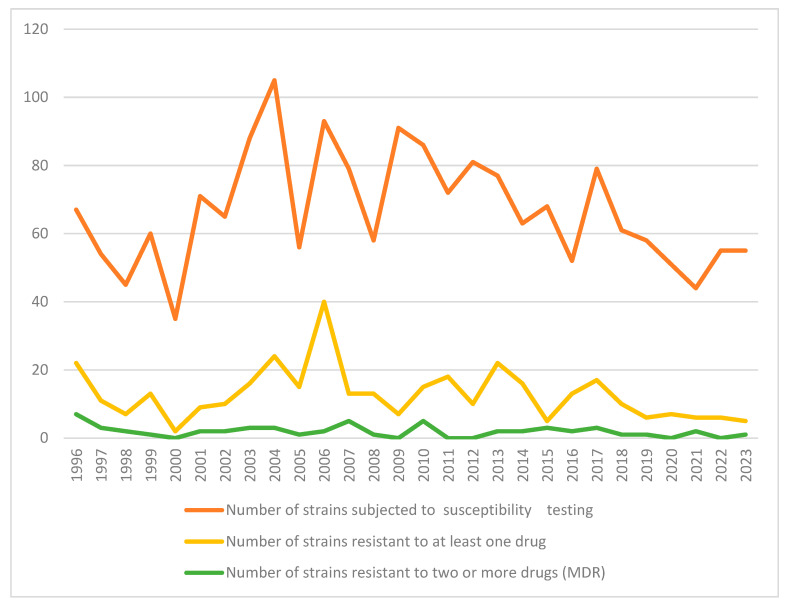
Annual distribution of any drug resistance and MDR in *M. tuberculosis* strains.

When the proportion of strains resistant to at least one drug was examined by year, a decreasing trend was observed between 2000 and 2003 compared to the previous period (1996–2002), increasing in the period 2004–2007 and decreasing again in the period 2008–2011 (*p* = 0.008) (Table 4). When the data were grouped and examined in 4-year periods, a statistically significant decreasing trend was found in the proportion of MDR strains over time (Table 4, Figure 2).

When the resistance rates for INH, RIF, ETM and SM were calculated by taking into account the cases showing resistance to one or more drugs, the highest resistance rate was observed against INH, at 12.6%; as expected, INH resistance was found to be the highest. This was followed by SM with 9.0%, ETM with 3.9% and RIF with 3.3%. The MDR rate was determined to be 2.9% (54 strains) (Table 5). A total of 15 cases (0.8%) resistant to INH+RIF+ETM+SM drugs were detected. Although this situation does not technically fully meet the definition of XDR-TB, it is important in terms of showing that MDR-TB strains exhibit a broader resistance profile. The data of these cases are presented in detail in Table 5. Since susceptibility tests were not performed for fluoroquinolones and second-line drugs such as amikacin, capreomycin or kanamycin in our study, it could not be definitively determined whether these 15 cases could be classified as XDR-TB.

When the change in resistance to anti-TB drugs was examined over time, no significant trend was observed for RIF, ETM and SM, while a statistically significant decreasing trend was observed in the most common INH resistance (Table 6 and Table 7).

## 4. Discussion

With the rapid emergence of methods for diagnosing TB and their increasing availability worldwide, the rate of early diagnosis has increased. Successful diagnostic and therapeutic practices, such as the direct observation, follow-up, and treatment of identified patients and others in contact with them, have led to a decrease in the incidence of TB globally, but TB continues to be a major public health problem worldwide [12,13].

The increasing resistance to TB drugs and the development of MDR-TB are among the most important challenges in achieving global TB control. The treatment of patients with MDR-TB is difficult and requires the use of drugs that are less effective, more toxic, and more expensive than those used in first-line treatment.

Although many studies have been conducted on TB incidence and drug resistance in Türkiye, comprehensive studies are needed in terms of both the number of isolates and the duration of the study to obtain data from which reliable conclusions can be drawn about temporal trends. In this study, the susceptibility results of a total of 1878 strains were evaluated by removing repeated patient samples from 2752 *M. tuberculosis* complex strains that were found to be positive in a total of 104,598 samples analyzed in our university’s mycobacteriology laboratory over a period of 28 years and whose anti-TB susceptibilities were studied.

Within the scope of the “Direct Observation Treatment Strategy” implemented in the public health system in Türkiye since 2006, data on all TB patients nationwide are recorded and patients are followed up individually. In this way, the number and location of cases can be monitored, and the decreasing trend in TB and resistance rates can be followed [14,15,16]. In studies in Türkiye, the *M. tuberculosis* complex isolation rates vary between 4.9% and 13.8% [17,18,19,20,21,22,23,24,25,26,27,28,29,30,31,32,33,34,35,36,37,38,39,40,41,42,43]. In our study, the isolation rate in all samples was found to be lower, at 2.6% (1.5–4.8%) (Table 1 and Table 2). It is thought that the culture positivity rate is low due to the evaluation of a large sample sent with clinical suspicion in our study. Since we are a tertiary university hospital, samples of patients from our province and neighboring provinces whose diagnosis has not yet been confirmed but who have received a preliminary diagnosis of TB are studied in our center. This situation causes undiagnosed TB cases to be included in the population, which naturally leads to a lower culture positivity rate. In conclusion, the fact that our study covers a large and heterogeneous population may have resulted in the culture positivity rate differing from the general population average.

Considering the national data in Türkiye, approximately two-thirds of TB cases were pulmonary TB cases throughout all years [43]. This situation is consistent with the fact that 1878 (68.7%) of the 2732 samples in which culture positivity was detected in our study were of pulmonary origin (Table 3 and Table 4).

In our study, it was determined that the positivity rate in pulmonary samples decreased from 16.4% in the 1996–1999 period to 4.7% in the 2020–2023 period (Table 2). This remarkable decrease can be explained by the successful TB control strategies implemented throughout Türkiye and especially in our province, and the effectiveness of directly observed treatment (DGT) applications.

On the other hand, although it is seen that the positivity rates in extrapulmonary samples generally decreased over time, a statistically significant increase is striking in the 2004–2007 period (*p* < 0.001, Table 2, Figure 1). During this period, the extrapulmonary positivity rate increased to 6.1%. During this period, an increased awareness of extrapulmonary TB in immunocompromised individuals (e.g., HIV-positive patients, organ transplant recipients, long-term corticosteroid users) may have led to the more frequent investigation of these cases and thus a temporary increase in diagnosis rates. In general, these findings further demonstrate the importance of following and examining changes in TB epidemiology over time.

Of the 1869 *M. tuberculosis* strains whose susceptibility to anti-TB drugs was analyzed, 19.2% were found to be resistant to at least one drug. This rate fluctuated over time, while a significant decreasing trend was observed in MDR (Table 5, Table 6 and Table 7). When similar studies conducted in Türkiye were examined, it was observed that MDR-TB rates decreased over time, similar to our study [43].

The fluctuating trend between the periods in question may be due to changes in many factors over time, such as the rational use of antibiotics affecting the development of antimicrobial resistance and surveillance programs implemented to monitor resistant strains. In addition, the statistically significant decrease in the rate of MDR strains detected on a four-year basis in our study suggests that long-term intervention strategies may be effective in controlling resistance. These findings emphasize the need for sustainable, multifaceted and interdisciplinary approaches to combating antimicrobial resistance.

The rate of susceptibility to first-line drugs varies in different regions of the world, including Türkiye. Studies on these rates conducted in different regions of Türkiye are summarized in Table 8.

When Table 8 is examined, it can be seen that there are significant fluctuations in drug resistance rates in *M. tuberculosis* strains isolated from different regions of Türkiye according to years and provinces. These fluctuations may be due not only to epidemiological changes but also to different reasons such as diagnostic methods and reporting processes. For example, while INH resistance was 11.9% in İzmir in 1999, this rate decreased to 4.2% in the 2002–2003 period. This decrease may be related to changes in patient populations in the relevant center during that period. Similarly, while the INH resistance reported in Edirne was 9.0% in 2003, it increased to 27.1% in 2004. This increase may be related to the implementation of a more active surveillance program or changes in laboratory studies.

It can be observed that the INH, RIF and MDR rates have changed over time in three studies conducted in different years in İzmir province. In 1999, the MDR rate was 6.6%, decreased to 5.8% in 2001, but increased to 8.2% in the 2002–2003 period. In a study conducted by Karabay et al. [26], in Edirne in 2004, the MDR rate was found to be quite high at 11.6%. Similarly, high MDR rates are also noted in the provinces of Trabzon (14.7%) and Mersin (10.7%). The high rate of drug resistance in these regions may be due to problems in diagnosis and treatment processes or inappropriate treatment regimens. It is also striking that SM resistance is particularly high in some regions (e.g., Edirne 2004: 29.0%) [26].

On the other hand, MDR rates are observed to be below 2% in some provinces such as Balıkesir, Erzurum, Adana and Samsun. This situation may indicate differences in patient populations as well as effective surveillance systems and successful treatment programs. In the case of Balıkesir, INH resistance is 7.9% and RIF resistance is only 1.0%; these rates are below the national average.

In addition, the data from this study conducted in our country show that there has been a general decreasing trend in drug resistance in recent years. For example, INH and RIF resistance are lower in most studies conducted after 2010. This suggests that control programs such as the Directly Observed Treatment Strategy (DOTS) implemented in Türkiye are effective.

However, some limitations should be taken into consideration when evaluating these data. The fact that the data were collected in different years and centers may create differences in standard methods. In addition, the sample size is low in some provinces (e.g., Düzce *n* = 62, Mardin *n* = 81), which reduces generalizability.

As a result, TB drug resistance in Türkiye shows a heterogeneous distribution regionally and over time; monitoring these rates with continuous surveillance systems at the national level constitutes an important goal in planning the measures to be taken. When the results of our study and these studies are evaluated together, it can be said that although there are regional differences in the rates of resistance to anti-TB drugs, INH resistance rates are the highest in our country (Table 8) [17,18,19,20,21,22,23,24,25,26,27,28,29,30,31,32,33,34,35,36,37,38,39,40,41,42,43].

We believe that the results obtained in our study may reflect the TB drug resistance patterns in the Southern Marmara region of Türkiye and may be a valuable guide for epidemiologists nationwide. The large number of samples included in this study (104,598 samples) and the long period (28 years) increase the reliability and generalizability of the findings. In addition, the fact that the study was conducted in a tertiary health center that not only serves the city of Bursa but also accepts patients from surrounding provinces strengthens the representativeness of the data beyond the local level, potentially at regional and national scales.

Defining drug resistance patterns and continuously monitoring drug-resistant TB are critical steps in reducing resistance rates. Therefore, long-term local surveillance studies such as this are extremely important. Data obtained from such studies may contribute not only to regional health policy planning but also to national and even international TB epidemiology [9,43]. In this context, we believe that the findings of our study may provide valuable insights for TB drug resistance surveillance in Türkiye and help shape future control strategies.

There are some limitations of this study that should be noted here. The retrospective nature of this study is a limitation as it prevents the classification of resistant cases as previously treated and relapsed. Conducting multicenter studies by adding information about the clinical status and treatment of patients in similar studies will make significant contributions to the fight against TB.

## 5. Conclusions

The resistance rates of *M. tuberculosis* complex strains to primary anti-TB drugs are consistent with the average resistance rates reported by the Tuberculosis Control Department of the Turkish Ministry of Health. This study is important in terms of reflecting the *M. tuberculosis* complex drug resistance patterns in our region and also showing that successful diagnostic and therapeutic practices such as the direct observation, follow-up and treatment of detected patients and other people who come into contact with them lead to a decrease in TB cases and resistance rates in our country and worldwide. Although our study was conducted as a single-center study, our center is a regional hospital that accepts patients not only from the province where it is located but also from many neighboring provinces. Therefore, the samples evaluated in this study have the potential to reflect not only local but also regional resistance patterns. However, we believe that the findings should be supported by multicenter studies in order to be generalizable at a broader level.

In addition, it should be noted that changes in diagnostic methods may affect the observed resistance and positivity rates. In our study, the results of molecular diagnostic and resistance analysis methods, which have been used in our institution in recent years, were not evaluated. When compared with culture, it is possible that these methods may change the number of cases and therefore affect the positivity and resistance rates. Therefore, we believe that these new methods should be included in future studies and that the effects of possible changes in the diagnostic methods used should be taken into account.

## Figures and Tables

**Table 1:** Distribution of specimens with positive culture tests between 1996 and 2023.

Year	Pulmonary Samples			Extrapulmonary Samples			Total Sample Number	Total Positive Sample Number (%)
	Sample Number	Positive Sample Number (%)	*p*	Sample Number	Positive Sample Number (%)	*p*		
1996	1105	64 (5.8)	**<0.001**	904	32 (3.5)	**<0.001**	2009	96 (4.8)
1997	1492	57 (3.8)		1179	32 (2.7)		2671	89 (3.3)
1998	1671	100 (6.0)		1558	25 (1.6)		3229	125 (3.9)
1999	1502	85 (5.7)		1456	24 (1.6)		2958	109 (3.7)
**1996–1999**	**5770**	**306 (5.3)**		**5097**	**113 (2.1)**		**10,867**	**419 (3.9)**
2000	1462	29 (2.0)		1102	18 (1.6)		2564	47 (1.8)
2001	1935	60 (3.1)		1181	23 (1.9)		3116	83 (2.7)
2002	1810	47 (2.6)		1199	26 (2.2)		3009	73 (2.4)
2003	1633	71 (4.3)		1138	28 (2.5)		2771	99 (3.6)
**2000–2003**	**6840**	**187 (2.7)**		**4620**	**95 (2.1)**		**11,460**	**302 (2.6)**
2004	1599	64 (4.0)		1613	41 (2.5)		3212	105 (3.3)
2005	2127	45 (2.1)		2486	26 (1.0)		4613	71 (1.5)
2006	1374	47 (3.4)		2322	55 (2.4)		3696	102 (2.8)
2007	1231	40 (3.2)		1982	71 (3.6)		3213	111 (3.5)
**2004–2007**	**6331**	**196 (3.1)**		**8403**	**193 (2.3)**		**14,734**	**389 (2.6)**
2008	1772	49 (2.8)		2557	20 (0.8)		4329	69 (1.6)
2009	2548	89 (3.5)		2840	17 (0.6)		5388	106 (2.0)
2010	1369	64 (4.7)		1927	30 (1.6)		3296	94 (2.9)
2011	1783	49 (2.7)		1963	25 (1.3)		3746	74 (2.0)
**2008–2011**	**7472**	**251 (3.4)**		**9287**	**92 (1.0)**		**16,759**	**343 (2.0)**
2012	1918	71 (3.7)		1476	15 (1.0)		3394	86 (2.5)
2013	2281	95 (4.2)		1492	47 (3.2)		3773	142 (3.8)
2014	2668	90 (3.4)		1632	24 (1.5)		4300	114 (2.7)
2015	2591	95 (3.7)		1388	27 (1.9)		3979	122 (3.1)
**2012–2015**	**9458**	**351 (3.7)**		**5988**	**113 (1.9)**		**15,446**	**464 (3.0)**
2016	1700	50 (3.0)		1675	12 (0.7)		3375	62 (1.8)
2017	1749	80 (4.6)		1875	58 (3.1)		3624	138 (3.8)
2018	1532	77 (5.0)		1360	40 (2.9)		2892	117 (4.0)
2019	2505	66 (2.6)		2297	36 (1.6)		4802	102 (2.1)
**2016–2019**	**7486**	**273 (3.6)**		**7207**	**146 (2.0)**		**14,693**	**419 (2.9)**
2020	1708	65 (3.8)		1791	22 (1.2)		3499	87 (2.5)
2021	1909	42 (2.2)		2414	28 (1.2)		4323	70 (1.6)
2022	3022	64 (2.1)		2684	36 (1.3)		5706	100 (1.8)
2023	4536	123 (2.7)		2575	36 (1.4)		7111	159 (2.2)
**2020–2023**	**11,175**	**294 (2.6)**		**9464**	**122 (1.3)**		**20,639**	**416 (2.0)**
**Total**	**54,532**	**1858 (3.4)**		**50,066**	**874 (1.7)**		**104,598**	**2752 (2.6)**

**Table 2:** Changes in culture positivity over four-year periods.

	Pulmonary Samples				Extrapulmonary Samples			
Years	Number of Culture Negative Samples	Number of Culture-Positive Samples	Odds Ratio	*p*	Number of Culture Negative Samples	Number of Culture-Positive Samples	Odds Ratio	*p*
1996–1999	5464	306	1.000	**<0.001**	4984	113	1.000	**<0.001**
2000–2003	6633	207	0.557		4525	95	0.926	
2004–2007	6135	196	0.570		8210	193	1.037	
2008–2011	7221	251	0.621		9195	92	0.441	
2012–2015	9107	351	0.688		5875	113	0.848	
2016–2019	7213	273	0.676		7061	146	0.912	
2020–2023	10.881	294	0.482		9342	122	0.576	

**Table 3:** Annual distribution of anti-TB drug susceptibility patterns of 1869 *M. tuberculosis* Strains.

Year	Number of Strains Subjected to Susceptibility Testing	Number of Strains Resistant to at Least One Drug (%)	*p*	Number of Strains Resistant to Two or More Drugs (MDR) (%)	***p***
1996	67	22 (32.8)	**0.008**	7 (10.4)	**0.025**
1997	54	11 (20.4)		3 (5.6)	
1998	45	7 (15.6)		2 (4.4)	
1999	60	13 (21.7)		1 (1.7)	
**1996–1999**	**226**	**53**		**13**	
2000	35	2 (5.7)		0	
2001	71	9 (12.7)		2 (2.8)	
2002	65	10 (15.4)		2 (3.1)	
2003	88	16 (18.2)		3 (3.4)	
**2000–2003**	**259**	**37**		**7**	
2004	105	24 (27.3)		3 (2.9)	
2005	56	15 (26.8)		1 (1.8)	
2006	93	40 (43.0)		2 (2.2)	
2007	79	13 (16.5)		5 (6.3)	
**2004–2007**	**333**	**92**		**11**	
2008	58	13 (22.4)		1 (1.7)	
2009	91	7 (7.7)		0	
2010	86	15 (17.4)		5 (5.8)	
2011	72	18 (25.0)		0	
**2008–2011**	**307**	**53**		**6**	
2012	81	10 (12.3)		0	
2013	77	22 (28.6)		2 (2.6)	
2014	63	16 (25.4)		2 (3.2)	
2015	68	5 (7.4)		3 (4.4)	
**2012–2015**	**289**	**53**		**7**	
2016	52	13 (25.0)		2 (3.8)	
2017	79	17 (21.5)		3 (3.8)	
2018	61	10 (16.4)		1 (1.6)	
2019	58	6 (10.3)		1 (1.7)	
**2016–2019**	**250**	**46**		**7**	
2020	51	7 (13.7)		0	
2021	44	6 (13.6)		2 (4.5)	
2022	55	6 (10.9)		0	
2023	55	5 (9.1)		1 (1.8)	
**2020–2023**	**205**	**24**		**3**	
**Total**	**1869**	**358 (19.2)**		**54 (2.9)**	

**Table 4:** Distribution of resistant strains in four-year periods.

		Resistant to at Least One Drug Strains			MDR Strains		
Years	Number of Strains	Number of Strains (%)	Odds Ratio	*p*	Number of Strains (%)	Odds Ratio	*p*
1996–1999	226	53 (23.5)	1.000	**0.008**	13 (5.8)	1.000	**0.025**
2000–2003	259	37 (14.3)	0.544		7 (2.7)	0.455	
2004–2007	333	92 (27.6)	1.246		11 (3.3)	0.560	
2008–2011	307	53 (17.3)	0.681		6 (2.0)	0.327	
2012–2015	289	53 (18.3)	0.733		7 (2.4)	0.407	
2016–2019	250	46 (18.4)	0.736		7 (2.8)	0.472	
2020–2023	205	24 (11.7)	0.433		3 (1.5)	0.243	

**Table 5:** Distribution and resistance rates of *M. tuberculosis* strains found to be resistant to anti-TB drugs.

Drugs	Number of Strains Resistant to Anti-TB Drug/Drugs	%
INH	112	5.9
RIF	7	0.4
ETM	26	1.4
SM	86	4.6
INH+RIF	19	1.0
INH+ETM	12	0.6
INH+SM	53	2.8
RIF+ETM	1	0.05
RIF+SM	0	0
ETM+SM	2	0.1
INH+RIF+ETM	12	0.6
INH+RIF+SM	8	0.4
INH+ETM+SM	5	0.3
RIF+ETM+SM	0	0
INH+RIF+ETM+SM	15	0.8

**Table 6:** Four-year distribution of anti-TB drug resistance rates.

Years	1996–1999	2000–2003	2004–2007	2008–2011	2012–2015	2016–2019	2020–2023
**Number of strains studied**	226	259	333	307	289	250	205
**INH**	27	13	24	8	23	9	8
**RIF**	1		1	2		3	
**ETM**	1	4	5	8	3	5	
**SM**	5	5	25	15	14	14	8
**INH+RIF**	7	3	1		2	4	2
**INH+ETM**		3	7		2		
**INH+SM**	4	4	16	14	4	6	5
**RIF+ETM**						1	
**RIF+SM**							
**ETB+SM**			2				
**INH+RIF+ETM**	5	2	1	1	2	1	
**INH+RIF+SM**		1	3	1	1	1	1
**INH+ETM+SM**	2	1	1			1	
**RIF+ETM+SM**							
**INH+RIF+ETM+SM**	1	1	6	4	2	1	

**Table 7:** Change in isoniazid resistance over four-year periods.

Years	Number of INH Resistant Strains	Number of INH Susceptible Strains	Odds Ratio	*p*
1996–1999	27	199	1.000	**0.002**
2000–2003	13	246	0.389	
2004–2007	24	309	0.572	
2008–2011	8	299	0.197	
2012–2015	23	266	0.637	
2016–2019	9	241	0.275	
2020–2023	8	197	0.299	

**Table 8:** Anti-TB sensitivity results in various studies in Türkiye [17,18,19,20,21,22,23,24,25,26,27,28,29,30,31,32,33,34,35,36,37,38,39,40,41,42].

Province	Year(s) of Isolation	n	INH (%)	RIF (%)	ETM (%)	SM (%)	MDR (%)
Şenol G/İzmir	1999	2691	11.9	10.2	11.9	1.9	6.6
Sürücüoğlu S/Manisa	1997–2003	355	16.9	9.0	9.8	14.9	7.3
Sayğan MB/Ankara	1999–2002	505	13.3	13.3	3.4	9.1	7.9
Saral ÖB/Trabzon	1998–2004	442	24.6	15.8	18.8	9.9	14.7
Şenol G/İzmir	2001	2393	9.9	9.3	6.4	8.6	5.8
Şenol G/İzmir	2002–2003	470	4.2	1.2	0.2	4.4	8.2
Aslan G/Mersin	2002–2003	127	15.0	15.0	7.5	7.5	5.0
Tansel Ö/Edirne	2003	134	9.0	4.5	2.2	1.5	3.0
Öztürk CE/Düzce	2000–2004	62	8.0	4.8	0.0	11.3	4.8
Durmaz R/Malatya	2000–2004	145	13.5	5.3	5.3	13.5	4.8
Karabay O/Edirne	2004	214	27.1	21.5	10.3	29.0	11.6
Gönlügür U/Sivas	2004–2006	158	17.7	4.4	5.1	11.4	3.8
Aslan G/Mersin	2005–2006	84	33.3	22.6	14.3	17.8	10.7
Yılmaz A/Erzurum	2015–2019	419	11.9	4.1	3.6	11.7	3.6
Özen N/Balıkesir	2011–2019	1004	7.9	1.0	2.8	4.6	1.0
Özmen E/Erzurum	2014–2016	120	9.2	3.3	0.2	5.8	1.7
Kayhan S/Samsun	2005–2010	1607	10.2	1.0	1.7	2.7	3.9
Öner O/Edirne	2016–2017	120	8.3	3.3	3.3	7.5	4.2
Behçet M/Bolu	2008–2018	138	10.1	4.3	2.9	12.3	2.9
Yazısız H/İstanbul	2011–2012	974	20.2	8.4	6.5	14.4	7.0
Arslan N/İzmir	2013–2019	321	6.8	2.2	0.6	7.5	0.6
Selek MB/	2010–2016	252	20,6	7.5	6.7	12.3	7.1
Alışkan HE/Adana	2005–2010	373	3.2	2.1	0.5	2.9	2.1
Öncel B/İstanbul	2011–2017	251	20.0	5.2	8.2	9.6	4.0
Terzi HA/Sakarya	2012–2017	466	9.8	4.1	4.0	7.7	4.1
Aksu M/Mersin	2010–2014	244	20.1	6.6	4.1	11.1	5.7
Etiz P/Adana	2013	123	13.5	1.8	2.7	8.1	1.8
Özcan N/Diyarbakır	2012–2015	415	21.4	6.3	6.7	15.7	5.1
Mardin, Kabak M/Mardin	2012–2018	81	13.6	6.2	6.2	4.9	0.0

## Data Availability

All data and materials are available and the corresponding author is Payaslıoğlu M.
